# Valuable Hepatoprotective Plants - How Can We Optimize Waste Free Uses of Such Highly Versatile Resources?

**DOI:** 10.3389/fphar.2021.738504

**Published:** 2021-11-18

**Authors:** Lubov V. Krepkova, Aleksandra N. Babenko, Olga L. Saybel’, Irina A. Lupanova, Olga S. Kuzina, Kathleen M. Job, Catherine M. Sherwin, Elena Y. Enioutina

**Affiliations:** ^1^ Center of Medicine, All-Russian Research Institute of Medicinal and Aromatic Plants (VILAR), Moscow, Russia; ^2^ Division of Clinical Pharmacology, Department of Pediatrics, University of Utah School of Medicine, Salt Lake City, UT, United States; ^3^ Department of Pediatrics, Boonshoft School of Medicine, Dayton Children’s Hospital, Wright State University, Dayton, OH, United States; ^4^ Department of Pharmaceutics & Pharmaceutical Chemistry, College of Pharmacy, University of Utah, Salt Lake City, UT, United States

**Keywords:** Milk Thistle, Chicory, Artichoke, rational use, food, phytodrug, functional food, animal feed

## Abstract

Humans used plants for thousand of years as food, drugs, or fuel to keep homes warm. People commonly used fruits and roots, and other parts of the plant were often wasted. This review aims to discuss the potential of rational stem-to-stern use of three highly versatile and valuable plants with hepatoprotective properties. Milk thistle (*Silybum marianum* L. Gaertn.), artichoke (*Cynara cardunculus*), and chicory (*Cichorium intybus* L.) have well-characterized hepatoprotective properties. These plants have been chosen since liver diseases are significant diseases of concern worldwide, and all parts of plants can be potentially utilized. Artichoke and chicory are commonly used as food or dietary supplements and less often as phytodrugs. Various dietary supplements and phytodrugs prepared from milk thistle (MT) fruits/seeds are well-known to consumers as remedies supporting liver functions. However, using these plants as functional food, farm animal feed, is not well-described in the literature. We also discuss bioactive constituents present in various parts of these plants, their pharmacological properties. Distinct parts of MT, artichoke, and chicory can be used to prepare remedies and food for humans and animals. Unused plant parts are potentially wasted. To achieve waste-free use of these and many other plants, the scientific community needs to analyze the complex use of plants and propose strategies for waste-free technologies. The government must stimulate companies to utilize by-products. Another problem associated with plant use as a food or source of phytodrug is the overharvesting of wild plants. Consequently, there is a need to use more active cultivation techniques for plants.

## Introduction

Humans have been using plants as food and remedies for thousands of years. About 80% of the world’s population uses medicinal plants and phytodrugs to treat various diseases (Ekor, 2014). Based on ethnopharmacological studies conducted by Sun et al., plants have a critical role in people’s diets, with roots and fruits most commonly used. Currently, wild and cultivated plants are used by the food, pharmaceutical, and cosmetic industries (Schmidt, 2012; Nofal et al., 2019; Rahman et al., 2019). The demand for wild plants, specially harvested in ecologically clean areas, is growing. Therefore, overharvesting of wild plants can lead to their endangerment (United Plant Savers, 2021). Often distinct parts of the plant are used as food or medicine. This raises the question of what happens to by-products and whether it is possible to develop waste-free processes of plant handling. Parts of the plants that may be wasted can be used as a source of food, functional food, or feed for farm animals. Therefore, researchers urge companies to introduce ethically reasonable use of plants.

According to the encyclopedia Britannica, food is a “substance consisting essentially of protein, carbohydrate, fat, and other nutrients used in the body of an organism to sustain growth and vital processes and to furnish energy” (Britannica Encyclopaedia., 2020). The Japanese government first introduced the term “functional food” in the early 1980s (Martirosyan and Singh, 2015). Later functional food was introduced to the European and American markets (Martirosyan and Singh, 2015). A function food “may provide a health benefit beyond the traditional nutrients it contains” (Thomas and Earl, 1994). In 1994, a new category of natural products, a dietary supplement, was introduced to consumers (1994). The Dietary Supplement Health and Education Act (DSHEA) describes dietary supplements as preparations intended to supplement the diet and may contain plants, vitamins, minerals, amino acids, tissue from organs, enzymes, and probiotics (1994). Dietary supplements are usually minimally regulated by medical authorities. According to the United States Food and Drug Administration (US FDA), about 50% of United States adults regularly use medicinal plants/herbs (Gottlieb, 2019). Phytodrugs, another category of natural products, are purified extracts from various parts of the plant or single-molecule phytochemicals isolated from natural products. These products are tightly regulated by regulatory authorities worldwide (Enioutina et al., 2020). The US FDA considers the highly purified extracts prepared from medicinal plants as botanical drugs and similarly regulates them as conventional synthetic drugs (Enioutina et al., 2020).

The primary purpose of this article is to discuss the potential of rational “stem-to-stern” use of plants. To prove the potential of rational use, we have chosen three plants with confirmed hepatoprotective properties. These plants are milk thistle (MT, *Silybum marianum* L. Gaertn.), artichoke (*Cynara cardunculus*), and Chicory (*Cichorium intybus* L.) (Baginskaya et al., 2000; Abenavoli et al., 2010; Li et al., 2014; Ben Salem et al., 2019; Achufusi and Patel, 2020; Mukhtar et al., 2021; Perovic et al., 2021; Song et al., 2021). The term “hepatoprotective” is often used to describe the ability of a drug or plant to prevent liver damage, whereas “antihepatotoxic” drugs can prevent or treat liver damage done by hepatotoxic substances. In general, these terms are often used interchangeably. In this article, we have chosen to use the term “hepatoprotective” plants.

The chief reason for selecting these plants is that liver disorders, including cirrhosis, viral hepatitis, hepatocellular carcinoma, and drug-, heavy metal-, and alcohol-induced liver injuries, are significant diseases of concern worldwide (Rehm et al., 2013; Asrani et al., 2019; Bashir et al., 2021). One-third to nearly 90% of patients with liver diseases or cancer diagnoses used herbal products with hepatoprotective properties (Fenclova et al., 2019).

Another reason is that the consumers widely use these plants as food, food substitute, phytodrugs, or dietary supplements. Artichoke flowers are primarily used as food or dietary supplements, while wasted aerial parts have the potential to be used as phytodrug, animal feed, or biofuel (Fernández et al., 2006; Gostin and Waisundara, 2019; Barbosa et al., 2020). The artichoke is often added to the hepatoprotective proprietary blends along with MT. Chicory is also best known as food (e.g., coffee substitute) (Wu and Cadwallader, 2019) or dietary supplement (inulin, a prebiotic driven from chicory root) (Shoaib et al., 2016) and less known as a phytodrug. The chicory wasted aerial part is occasionally a part of the phytodrug and can be used as animal feed (Niness, 1999; Githiori et al., 2006; Kandeler and Ullrich, 2009; Himalaya Wellness Company., 2021b). Chicory is a part of a multi-component phytodrug developed by the Himalaya Drug Company (Himalaya Wellness Company., 2021a). Unlike the other two plants, milk thistle fruits/seeds are mainly used as phytodrugs or dietary supplements, but the remaining plant parts can also be used as food additives for animals and biofuel (Chabaev et al., 2011; Andrzejewska et al., 2015; Ataei Nukabadi et al., 2021). Several MT-based phytodrugs were marketed [e.g., Carsil (SoPharma, Bulgaria); and Legalon (Flordis, Australia)]. Currently, MT-based dietary supplements are in the top-40 best-selling herbal supplements (Smith et al., 2017).

Distinct parts of these plants have different compositions of nutrients and phytochemicals. For example, the bioactive constituents responsible for the hepatoprotective properties of MT are present in the highest concentration in plant fruits and seeds (Baginskaya et al., 2000; Vargas-Mendoza et al., 2014). The hepatoprotective phytochemicals are present in roots and aerial part of chicory (Thabrew et al., 1982; Huseini et al., 2005; Abenavoli et al., 2010; Cha et al., 2010; Abd El-Mageed, 2011).

The demand for phytodrugs and dietary supplements with hepatoprotective properties will grow. This may increase the exhaustion of wild-grown resources and waste parts of plants not used for drug/supplement preparation. This article will show the potential use of distinct parts of MT, artichoke, and chicory as food, phytodrugs, dietary supplements, functional food, and animal feed.

## Methods

This extensive review (Munn et al., 2018) of existing literature and evaluation of the existing literature found numerous gaps in our knowledge related to the use of distinct parts of MT, chicory, and artichoke in food and pharmaceutical industries animal husbandry. The authors conducted a comprehensive search in six electronic databases (PubMed, Embase, Scopus, ScienceDirect, Web Science, and elibrary.ru), the All-Russian Institute of Aromatic and Medicinal Plants (VILAR) medical library, and other relevant medicinal herbs websites for literature published between January 1976 and May 2021. Searches were conducted using the keywords: artichoke, chicory, *Silybum marianum*, *Cichorium intybus*, phytodrugs, drugs, dietary supplements, food, functional food, and animal feed.

## Milk Thistle

Milk thistle (MT, *Silybum marianum* L. Gaertn.,), a member of the Asteraceae family, is native to Southern Europe and Asia and widely naturalized in Europe, North America, South America, Australia, and New Zealand (Missouri Botanical Garden., 2019). It is not used actively in agriculture and is considered to be an invasive weed in North America, Australia, New Zealand, and South Africa (King County, 2018).

MT is best known for its hepatoprotective and antioxidant properties (Siegel and Stebbing, 2013) (Table 1). Bioactive compounds isolated from MT, specifically a bioactive flavonolignan, silymarin, have antioxidant properties, protect against damage by the free radicals generated from the metabolism of ethanol, acetaminophen, and carbon tetrachloride ingested in excessive amounts (Krepkova et al., 2008; Vargas-Mendoza et al., 2014). In addition, MT has anti-inflammatory, anti-cancer, antiviral, and immunomodulatory properties (Deak et al., 1990; Krepkova et al., 2008; Vargas-Mendoza et al., 2014) (Table 2). Carsil, Legalon, and Silimar are popular phytodrugs prepared from the fruit of MT (Brailski et al., 1986; Feher et al., 1989; Soleimani et al., 2019; Gillessen and Schmidt, 2020). Now, MT and plant extracts are immensely popular as dietary supplements (Table 1).

**TABLE 1:** The areas of usage of distinct parts of the plants.

Plant name	Part of the plant	Areas of usage
Milk thistle	Fruits (oilseed cake, oil, and seeds)	✓Phytodrugs Brailski et al. (1986); Baginskaya et al. (2000)
		✓Dietary supplements Smith et al. (2017); Fenclova et al. (2019).
		✓Human functional food & bakery products Semenkina (2010); Nekrasova and Popov (2020)
		✓Animal functional foodKolesnyk et al. (2009); Chabaev et al. (2011)
		✓Cosmetics Nofal et al. (2019)
		✓Biodiesel (fruit oil) Ullah et al. (2015)
	Aerial part	✓Animal feed Andrzejewska et al. (2015)
		✓Phytodrug Himalaya Wellness Company (2021b)
Artichoke	Flowers	✓Food Gostin and Waisundara (2019)
		✓Dietary supplements GistGear (2021)
		✓Cosmetics D'Antuono et al. (2018)
	Aerial part	✓Dietary supplements Zeaiter et al. (2019)
		✓Animal feed Barbosa et al. (2020)
		✓Biofuel Barbosa et al. (2020)
Chicory	Aerial part	✓Phytodrugs Himalaya Wellness Company. (2021a)
		✓Dietary supplements Perovic et al. (2021)
		✓Functional food Perovic et al. (2021)
		✓Animal feed Nwafor et al. (2017)
	Roots	✓Dietary supplements Shoaib et al. (2016)
		✓Biofuel Hughes et al. (2017)

**TABLE 2:** Phytochemical composition and pharmacological properties of milk thistle.

Plant name/part of the plant commonly used for medicinal purposes	Bioactive constituents	Pharmacological properties
**Milk thistle** *Silybum marianum* L. Gaertn./fruits and seeds 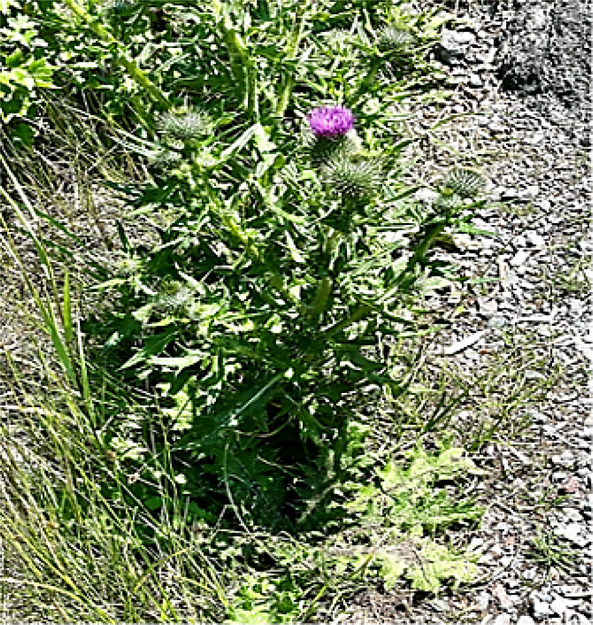	Silibinin 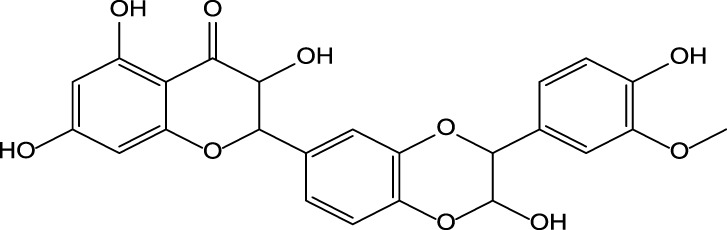 Silidianin 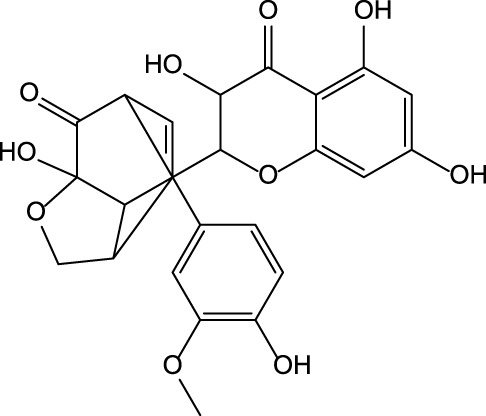 Silychristin 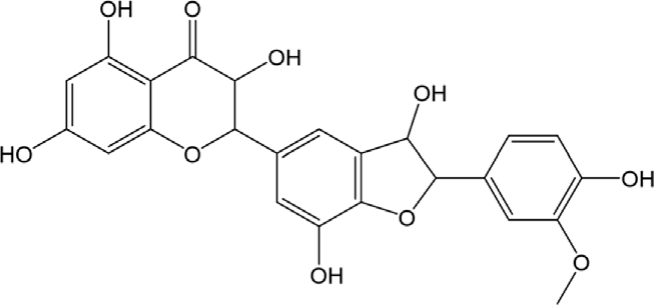	✓Hepatoprotective Flora et al. (1998); Baginskaya et al. (2000); Sokol’skaya (2000); Krepkova and Sokol’skaya (2007); Romanova and Krasavtsev (2007); Krepkova et al. (2008); Abenavoli et al. (2010); Schmidt (2012); Vargas-Mendoza et al. (2014) ✓Antioxidant Siegel and Stebbing (2013) ✓Anti-inflammatory Krepkova and Sokol’skaya (2007); Lee et al. (2007); Krepkova et al. (2008) ✓Anti-cancer Lee et al. (2013) ✓Immunomodulatory Deak et al. (1990)

However, the use of MT as food, functional food for humans and animals is not well studied. Several publications describe the usefulness of bread or pastry fortification with MT fruits and oil and the supplementation to animal feed with bioactive compounds from the plant. It appears that plant stems and foliage are not used following phytodrugs and dietary supplements preparations and have the potential to be wasted.

### Milk Thistle Bioactive Constituents and Their Biological Activity

Silymarin is the main bioactive constituent found in the MT fruit oilseed cake (Vargas-Mendoza et al., 2014) (Table 2). It is a complex flavonolignan responsible for the plant’s hepatoprotective properties. Silymarin is a complex flavonolignan comprising silybin A and B, isosilybin A and B, silydianin, silychristin, isosilychristin, and taxifolin, a flavonoid precursor (Pradhan and Girish, 2006; Vargas-Mendoza et al., 2014). The silibinin, a mixture of silybins A and B, is also named silibinin. The ratio of flavonolignan isomers may differ depending on the region where MT was grown (Lee et al., 2007; Poppe and Petersen, 2016). Flavonolignan’s concentrations can also be influenced by oil extraction technology from MT fruits. The cold-pressed oil extraction preserves a unique combination and activity of bioactive constituents present in the plant fruits (Hou et al., 2010; Lee et al., 2013). The MT oilseed cake or seeds are the primary raw material used by the pharmaceutical and dietary supplement companies to prepare phytodrugs and dietary supplements.

The hepatoprotective properties of silibinin include blocking stabilizing liver cell membranes and blocking phosphodiesterase (Romanova and Krasavtsev, 2007). In addition, silibinin can bind radicals due to its phenolic structure, resulting in a significant increase in the reduced glutathione content in the liver. This reduction in glutathione increases the organ’s protection from oxidative stress, maintaining its normal detoxification function.

Silymarin reduces inflammation via downregulation of the expression of transcription factors [nuclear factor-kappa B (NF-kB) and signal transducer and activator of transcription 1 (STAT-1)] and inflammation-associated proteins. The downregulation of transcription factors led to a significant reduction of interleukin 1 alpha (IL1α) and tumor necrosis factor-alpha (TNFα) production (Lee et al., 2013) (Table 2). It has also been reported that silibinin may suppress melanoma cell growth by reducing the phosphorylation of extracellular signal-regulated kinase (ERK)-1/2. The reduction of ERK-1/2 resulted in downregulation of the MEK1/2 and reduced activity of NF-κB and STAT-3, followed by the cell-cycle arrest in the G1 (Lee et al., 2013). Interestingly, silymarin, but not silibinin, could attenuate ischemia-reperfusion-induced brain injury (Hou et al., 2010). Silibinin also reduced chemotherapeutic drug resistance in human bladder cancer cell lines via NF-kB dependent and independent pathways.

### Milk Thistle Hepatoprotective Properties

Bioactive flavonoids have been used to treat hepatobiliary system diseases (Flora et al., 1998; Pradhan and Girish, 2006; Abenavoli et al., 2010) (Table 1). Flavonolignans isolated from the fruit of MT prevail in this group of drugs. Studies have shown that the hepatoprotective effect of these phytodrugs are due to the presence of three flavonolignans: silibinin, silidianin, and silicristin (Abenavoli et al., 2010). Numerous preclinical studies were published demonstrating hepatoprotective properties of MT or MT extracts in animal models of liver injury. Only the PubMed database holds 311 articles, including review articles, published between 1989 and 2021. Several animal models of hepatotoxicity were used by investigators, including acetaminophen, alcohol, carbon tetrachloride, phenylhydrazine, and thioacetamide (Abenavoli et al., 2010; Kantah et al., 2011; Ahmed et al., 2020).

It has been reported that liver fibrosis in rats induced by the 8-weeks intragastric administration of carbon tetrachloride (CCl4) was successfully treated with a standardized extract of the MT (Tsai et al., 2008). The extract was administered at a 200 mg/kg dose four times per week for 3 weeks. The treatment has significantly decreased aspartate aminotransferase (AST), alanine aminotransferase (ALT), and alkaline phosphatase (ALP) in the serum of experimental animals.

A study confirming the hepatoprotective activity of Silimar, an original phytodrug developed by the VILAR, was conducted on a model of experimental hepatitis in rats induced by a single subcutaneous administration of oil-based CCl_4_ (Baginskaya et al., 2000; Sokol’skaya, 2000). Silimar at a dose of 100 mg/kg was administered 5 days before CCl_4_ administration and continued 21 days after liver damage. The administration of CCl_4_ resulted in a significant elevation of the γ-glutamyl transferase (GGT), ALP, ALT, and AST compared with healthy controls (Baginskaya et al., 2000). Following chronic administration of Silimar resulted in significantly reduced levels of GGT, ALT, AST. In an *in vitro* experiment, Silimar suppressed induced lipid peroxidation, evidenced by reducing the amount of malondialdehyde by 15–41% compared to the control (Baginskaya et al., 2000).

It appears that MT extracts have low bioavailability (El-Gazayerly et al., 2014; Mukhtar et al., 2021). The treatment of rats receiving CCl_4_ with silymarin incorporated into phytosomes significantly decreased superoxide dismutase activities and glutamic pyruvic transaminase compared to the plant extract free of phytosomes (El-Gazayerly et al., 2014). Additionally, the treatment with encapsulated silymarin inhibited liver fibrosis induced in rats by administering 2 mg/kg acetaminophen for 2 weeks (Mukhtar et al., 2021). The treatment significantly decreased levels of ALT, AST, and ALP.

Several investigators have studied the pharmacokinetics of MT in healthy volunteers and patients with various liver diseases. The study published by Calani et al. investigated the bioavailability and metabolism of flavonolignans after a single administration of the water-soluble MT extract (Calani et al., 2012). After overnight fasting, the healthy volunteers consumed 8 g of the extract dissolved in the water in this study. The study showed that MT flavonolignans have low bioavailability (0.45%). Urinary excretion was studied following 48 h. Thirty-one metabolites of flavonolignans have been found in the urine. The most common metabolites were monoglucuronides followed by sulfate–glucuronides and diglucuronides. Zhu et al. investigated the pharmacokinetics of individual flavonolignans in humans following single or chronic administration of MT extract (Zhu et al., 2013). The authors determined that all investigated flavonolignans were rapidly absorbed and eliminated by the organism. Flavonolignan exposure was dose-dependent. The most prevalent flavonolignans detected in volunteers were silybin A and silybin B, followed by isosilybin B and isosilybin A. The pharmacokinetics of MT flavonolignans was significantly altered in patients infected with Hepatitis C virus and patients with non-alcoholic fatty liver disease (Abenavoli et al., 2018). High exposure to bioactive flavonolignans was observed in patients with live cirrhosis.

Several MT phytodrugs are marketed under different brand names (e.g., Silymarin, Legalon, Carsil, and Silibor). Legalon is a standardized phytodrug containing 70–140 mg silymarin (calculated as silibinin) in 86–186 mg of dry extract of the fruit (European OTC medicines and devices., 2020). The phytodrug is intended to treat acute and chronic hepatitis and cirrhosis. Legalon at a 25 mg/kg dose reduced lipid peroxidation and increased superoxide dismutase activity in hepatic tissues (Batakov, 2001). It has been reported that workers chronically exposed to toluene and/or xylene vapors receiving oral Legalon for 30 days had significant improvements in liver function and platelet counts compared with workers that did not receive this treatment (Szilard et al., 1988). Legalon-SIL^®^ has been used for intravenous treatment of patients infected with hepatitis C virus (HCV) and not responding to pegylated interferon/ribavirin treatment (Rutter et al., 2011). Most of the patients (85%) had undetectable HCV ribonucleic acid (RNA) after a 14-days course of Legalon-SIL^®^.

Many clinical trials were conducted to find the efficacy of MT extracts in humans with various liver diseases. The first trial that we were able to find was conducted in 1980 and investigated the effectiveness of the MT derivatives for the treatment of chronic hepatopathies (De Martiis et al., 1980). The first randomized placebo-controlled trial was performed in 1989 and investigated the efficacy of silymarin in patients with liver cirrhosis (Ferenci et al., 1989). The patients in this trial received 140 mg of silymarin three times a day for an extended period. It has been determined that the 4-years survival rate in the treatment group was significantly higher compared with patients receiving placebo. Another double-blind placebo-controlled clinical trial proved the efficacy of milk thistle for treating patients with acute hepatitis (El-Kamary et al., 2009). The therapy reduced symptoms of biliary retention such as jaundice and scleral icterus. Interestingly, unlike in animal experiments, the reduction of ALT and AST in human subjects was not significant (El-Kamary et al., 2009). The evaluation of the efficacy of silymarin for treatment of non-alcoholic steatohepatitis has shown that patients receiving 700 mg of silymarin for 48-weeks had comparable to control non-alcoholic fatty liver disease activity scores; however, silymarin-treated patients had significantly lower liver fibrosis (Wah Kheong et al., 2017).

A more detailed analysis of available clinical data confirming hepatoprotective properties of MT is available in the recently published articles by Abenavoli et al. (Abenavoli et al., 2018) and Marmouzi et al. (Marmouzi et al., 2021). The literature analysis led us to conclude that MT standardized extracts can improve liver function following several hepatic disorders, including chronic exposure to toxic chemicals, viral hepatitis, liver cirrhosis, and non-alcoholic liver steatosis. However, this treatment cannot cure the disease. In our opinion, this treatment can be combined with the standard of care therapies, especially when there is no improvement following conventional treatment.

### Milk Thistle as Food and MT-Based Functional Food

MT oil, oilseed cake, or seeds could fortify bakery products (Semenkina, 2010; Ataei Nukabadi et al., 2021) (Table 1). MT fruits contain a complex of biologically active substances (vitamins, minerals, flavonoids, a significant amount of dietary fiber, and amino acids) (Semenkina, 2010). The MT seed oil is enriched with omega-6 and omega-3 fatty acids, tocopherols, and carotenoids. The addition of MT in bakery products may increase the nutritional value by supplying extra proteins, linoleic acid, vitamin E, and calcium. The presence of flavonolignans in the MT oilseed cake could supply additional support for liver function. MT flavonolignans may help increase bone calcium absorption when added to bakery products combined with fat-free milk (Semenkina, 2010; Nekrasova and Popov, 2020). The addition of nettle leaf and MT seed powder to sponge cakes could reduce blood sugar levels (Ataei Nukabadi et al., 2021) and improve liver functions.

Andrzejewska with colleague suggested that whole MT plant can be used as food and cosmetics (Andrzejewska et al., 2015). According to the Edible Wild Food resource, “the young stalks, leaves, roots and flowers can be eaten” (Edible Wild Food., 2021). Mediterranean communities have been using MT young stems as food for centuries (Andrzejewska et al., 2015). Spanish natives have been adding aerial parts to salads or eating them cooked (Andrzejewska et al., 2015). Unfortunately, the reports on the use of MT as food or functional food for humans are limited and primarily focused on the use of plant fruit, not aerial parts, potentially due to the presence of spikes on the leaves. MT may be an active ingredient of the cream intended to treat melasma (Nofal et al., 2019).

### Milk Thistle as Animal Feed

The MT can also be used as a part of functional food for farm animals (Table 1). MT oilseed cake is enriched in crude proteins, dietary fiber, and fat (Stastnik et al., 2020). The seed cake contains ∼4% of flavonolignans (Stastnik et al., 2020). It has been reported that the addition of MT oilseed cake in the diet of lactating dairy cows in the amount of 25% of the required digestible protein increased the protein and fat content in the milk, improved its amino acid composition, and augmented the average daily milk yield (Chabaev et al., 2011). The administration of MT oilseed cake at a dose of 44 mg/kg/day to Ayrshire heifers during the first twenty-first months of their lives reduced the number of diseases caused by impaired liver function and metabolic processes by ∼35% and prevented mortality of the livestock from the first month of cattle life to the end first lactation (Kravainis et al., 2014). The supplementation of the sow diet with MT at the dose 100 mg/kg/day starting from 88 days of gestation to farrowing positively affected the fetal formation and the weight of newborn piglets (Kolesnyk and Bankovska, 2008). The addition of MT to the sow fodders prevented suckling pig’s mortality and increased the average daily gain by ∼19% (Kolesnyk et al., 2009). Chickens receiving MT extract at doses of 0.1; 1.0; 1.5 and 2.0 mg/kg had the average daily body weight gain during the rearing period by 1.1–5.3%, the European productivity index increased 2.4–10.2%, and feed consumption per 1 kg of the weight gain increased 1.6–4.8% compared to the control group (Bagno et al., 2020). Feeding MT oilseed cake in combination with ascorbic acid to broiler chickens reduced the concentration of lead and cadmium in meat by 2.72 and 2.08 times and increased its biological value (Mildzikhov et al., 2013). Adding oilseed cake to the hens’ feed increased egg production and egg size (Stastnik et al., 2020). Some studies reported no effect on chicken wellbeing (Stastnik et al., 2020). As shown above, the fortification of farm animals’ fodder with the MT oilseed cake has significantly improved animals’ survival.

It has been hypothesized that the aerial part of the plant can also be used to fortify farm animal feed (Andrzejewska et al., 2015). It has been reported that MT can be used as supplemental food in cattle and chicken (Bagno et al., 2020; Shemuranova and Garifullina, 2020). Unfortunately, the nutritional value of the MT areal part is lower than barley (Stastnik et al., 2020).

## Artichoke

*Cynara cardunculus* L. is a member of the Asteraceae family. There are three taxonomic variants of the *Cynara cardunculus* species: *Cynara cardunculus* L., var. *scolymus* (L.) Fiori (globe artichoke), *Cynara cardunculus* L., var. *altilis* DC. (cultivating cardoon) and *Cynara cardunculus* L., var. *sylvestris* (Lamk), Fiori (wild cardoon) (Gostin and Waisundara, 2019). The plant is native to the Mediterranean area, and nowadays, it is cultivated in other countries (Gostin and Waisundara, 2019). Artichoke prefers hot and dry climates and can live in an adverse environment. The artichoke immature flower heads have a bitter-sweet flavor and are part of the diet in many countries.

Artichoke bioactive constituents have antibacterial, anti-malarian, antiviral (Hep C virus), anti-inflammatory, antioxidative, hepatoprotective, and metabolic effects (Ben Salem et al., 2015; Elsebai et al., 2016; Villarini et al., 2021) (Table 3). Constituents isolated from artichoke (e.g., cynaropicrin) are involved in the regulation of the NF-κB pathway and downregulate inflammatory cytokines (Elsebai et al., 2016). An extract of the artichoke leaves has anti-hypercholesterolemic and antioxidative properties in an *in vivo* model of rats with high-fat diet-induced obesity (Ben Salem et al., 2019). Prophylactic use of artichoke leaf and flower head extracts may also protect against cancer development (Abdel-Moneim et al., 2021). The preparations from artichoke can be added to cosmetics intended for prophylaxis of skin photoaging (D'Antuono et al., 2018).

**TABLE 3:** Phytochemical composition and pharmacological properties of artichoke.

Plant name/part of the plant commonly used for medicinal purposes	Bioactive constituents		Pharmacological properties
**Artichoke** (*Cynara cardunculus* L.)/immature flower 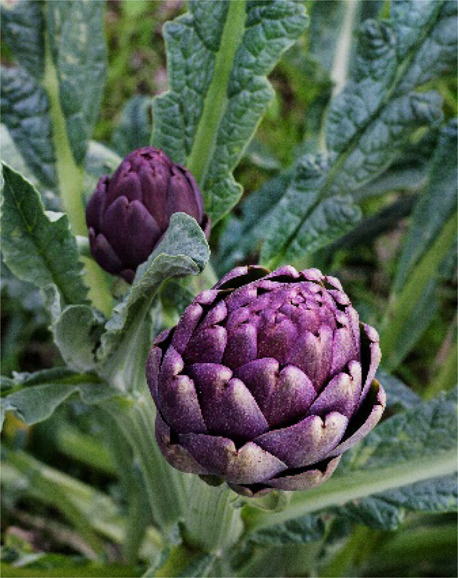	Luteolin 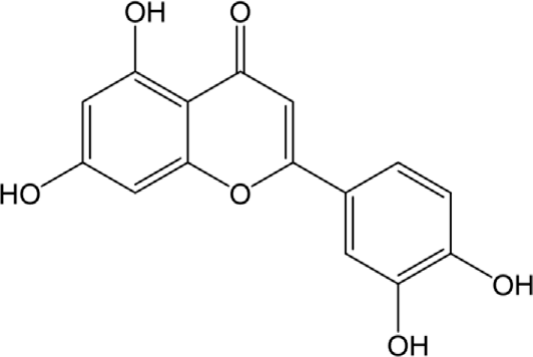	Luteolin 7-O-glucoside 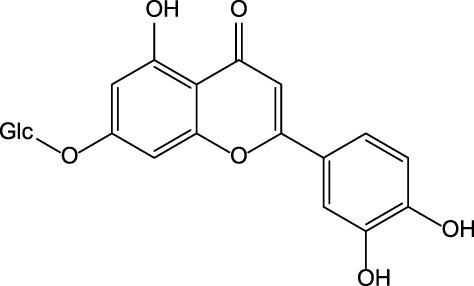	✓Hepatoprotective Ben Salem et al. (2015) ✓Anti-oxidant Ben Salem et al. (2015) ✓Anti-inflammatory Ben Salem et al. (2015) ✓Anti-cancer Abdel-Moneim et al. (2021); Villarini et al. (2021) ✓Anti-viral Elsebai et al. (2016) ✓Immunomodulatory ✓Hypolipidemic Bundy et al. (2008); Ben Salem et al. (2015)
	Caffeic acid 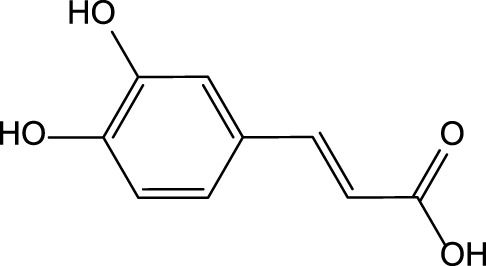	Chlorogenic acid 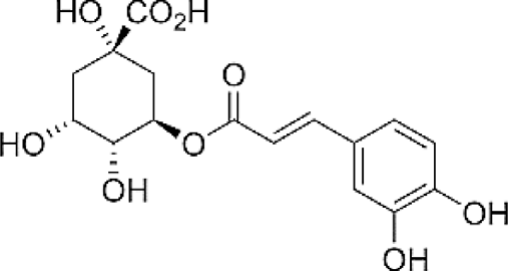	
	Cynarin 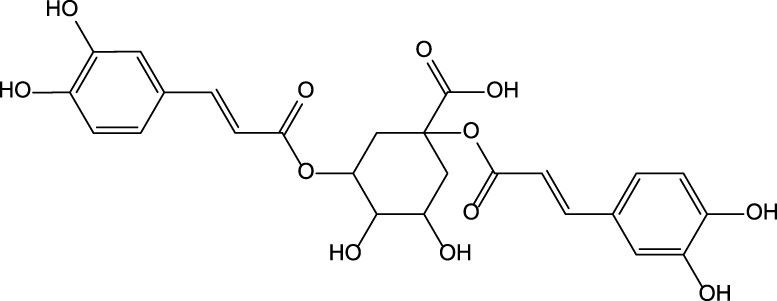		

Numerous companies market artichoke to improve liver function dietary supplement (Zeaiter et al., 2019; GistGear, 2021) (Table 1). The artichoke is often used in combination with MT and dandelion (GistGear, 2021). The immature flower is the most used part of the plant in the food industry. As a result, >80% of biomass can be wasted (Barbosa et al., 2020). The use of wasted artichoke biomass could produce phytodrugs, dietary supplements, biofuel, biodiesel, cellulose, and animal feed.

### Artichoke Bioactive Constituents and Their Biological Activity

The artichoke plant has significant levels of polyphenols, sesquiterpene lactones, terpenoids, carotenoids, and chlorophylls (Turkiewicz et al., 2019; Zeaiter et al., 2019; Rocchetti et al., 2020) (Table 3). The artichoke is an excellent source of inulin, minerals (e.g., potassium and phosphorus), and vitamins (vitamins group B and vitamin C) (Bundy et al., 2008; Zeaiter et al., 2019). Levels of phytochemicals can vary significantly depending on the taxonomic variant, cultivar, or hybrid plant (Turkiewicz et al., 2019). The artichoke bracts have elevated levels of inulin and polyphenols (Turkiewicz et al., 2019). The analysis of phytochemicals in the seed-propagated artichoke hybrids revealed that leaves of artichoke contain >90 polyphenols and >120 sesquiterpene lactones (Rocchetti et al., 2020). Seven cultivars were investigated by Rocchetti and others (Rocchetti et al., 2020). The authors reported significant differences in the composition of bioactive compounds present in these cultivars. As proved by Turkiewicz and others, the health-promoting benefits of artichoke depended on the levels of bioactive phytochemicals present in cultivars and hybrids (Turkiewicz et al., 2019).

The preclinical studies investigating pharmacological properties of the artichoke leaf compounds have found that the hepatoprotective properties of the phenolic compounds (e.g., luteolin, chlorogenic and caffeic acids, cynarin and luteolin 7-O-glucoside) are associated with their ability to reduce plasma levels of malondialdehyde induced by t-BHP and as a result decrease of free radicals and hepatocyte damage (Ben Salem et al., 2015) (Table 3). The phenolic leaf compounds also prevent lipid peroxidation. Additionally, phenolic compounds may exhibit hypocholesterolemic, anti-cholestatic, and antimicrobial effects (Ben Salem et al., 2015). The treatment of volunteers with total plasma cholesterol levels 6.0–8.0 mmol/l with artichoke leaf extract (ALE) significantly decreased total cholesterol levels, but not low-density lipoprotein and high-density lipoprotein (Bundy et al., 2008). An *in vitro* study demonstrated that treatment of HT-29 and RKO tumor cells with cynaropicrin, caffeoylquinic acids, and chlorogenic acid-induced cell apoptosis suggests that ALEs could be potentially used as an effective tool chemotherapeutic agent (Villarini et al., 2021) (Table 3).

### Artichoke Hepatoprotective Properties

The hepatoprotective properties of the artichoke were actively studied in animal models of acute and chronic liver injury (Tables 1, 3). In 1987, Adzed and others showed that cynarine and caffeic acid possess cytoprotective properties *in vitro* experiments (Adzet et al., 1987). The ethanolic ALE was given to experimental rats for 60 days, and a high-fat diet was significantly reduced by high-fat diet levels of ALT, AST, and ALP (Ben Salem et al., 2019). Another study reported that the ALE treatment of rats with diazinon-induced liver injury reduced levels of ALT, AST, ALT, and TNFα gene expression in hepatocytes (Ahmadi et al., 2019). The treatment of rats with ALE for 2 weeks improved ALT and AST levels after CCl_4_ hepatic injury and decreased DNA fragmentation and caspase 3 levels in liver tissues of CCl_4_-exposed rats (Colak et al., 2016). While most of the studies evaluating the hepatoprotective properties of the artichoke were done using leaf extracts. Sumer and others investigated the hepatoprotective properties of artichoke stems and bracts in an animal model of acetaminophen-induced liver injury in rats (Sumer et al., 2020). They found that stem and bract extract also effectively reduced ALT and AST levels but not ALP. It appears that the intensity of hepatoprotective effects of artichoke extracts is associated with the levels of phenolic compounds present in the extract (Speroni et al., 2003).

Artichoke extracts are marketed as phytodrugs for the treatment of liver disorders (Lattanzio et al., 2009). A double-blind clinical trial in patients with non-alcoholic liver injury confirmed the results of preclinical studies. Patients receiving ALE 600 mg daily for 2 months had an increased hepatic vein flow, reduced portal vein diameter, and improved lipid and hepatic enzyme profiles (Panahi et al., 2018). Another trial investigated the efficacy of a standardized ALE in patients with chronic Hep C infection (Huber et al., 2009). Unfortunately, the treatment of patients for 12 weeks with ALE 3,200 mg/day did not improve hepatic enzymes levels while reducing the patient’s fatigue and joint problems.

The reviewed literature confirmed that the artichoke extracts from leaves and other parts of the plant exhibit strong hepatoprotective properties (Table 1). More clinical trials are needed to verify the hepatoprotective properties of artichoke extracts in humans.

### Artichoke Use as Food or Functional Food

The artichoke was used as food from ancient times in Egypt, Greece, and Rome. Immature flowers and stems were used to prepare dishes, while mature flowers coagulate milk (Gostin and Waisundara, 2019) (Table 1). Modern-day artichoke flowers are exceedingly popular worldwide as a part of the everyday diet or delicatessen food. Immature flowers can be used fresh and canned or frozen for future use (Gostin and Waisundara, 2019). The artichoke was traditionally used in Mediterranean cuisine to prepare salads, dips, or soups (Barbosa et al., 2020). It can also be roasted with garlic and olive oil. The artichoke flowers and stigmas are used as a vegetable alternative to animal-driven rennet (Barbosa et al., 2020). The ability to clot milk is attributed to two enzymes mature artichoke flowers, cardosins A and B. The artichoke seed oil is enriched by linoleic, oleic, palmitic, and stearic fatty acids (Barbosa et al., 2020). The oil obtained from artichoke seeds is similar in composition to sunflower oil and can be used for human consumption and as a dietary supplement.

Artichoke is a rich source of non-digestible inulin, a heterogenous fructose polymer (Niness, 1999; Nwafor et al., 2017) and oligofructose, a shorter-chain oligomer, belonging to the inulin subgroup, which can be used as a part of dietary supplement blends or can be added to food to enhance its health benefits (Lattanzio et al., 2009). Inulin is well-known prebiotic that can support grows of a healthy intestinal microbiome. Antioxidant properties of phenolic compounds can be used in a functional food to support immune, endocrine, and cardiovascular systems (Table 3).

The biomass that is not used by the food industry could be a source of animal feed or feed supplement for farm animals, especially in areas of extensive artichoke cultivation. Silage artichoke (e.g., artichoke bracts and plants) has been investigated as an animal feed with generally positive results for animals and animal products (Meneses et al., 2007; Monllor et al., 2020) (Table 1). The analysis of artichoke foliage revealed that it is safe to use as animal feed.

### Artichoke By-Products Use

**FIGURE 1:**
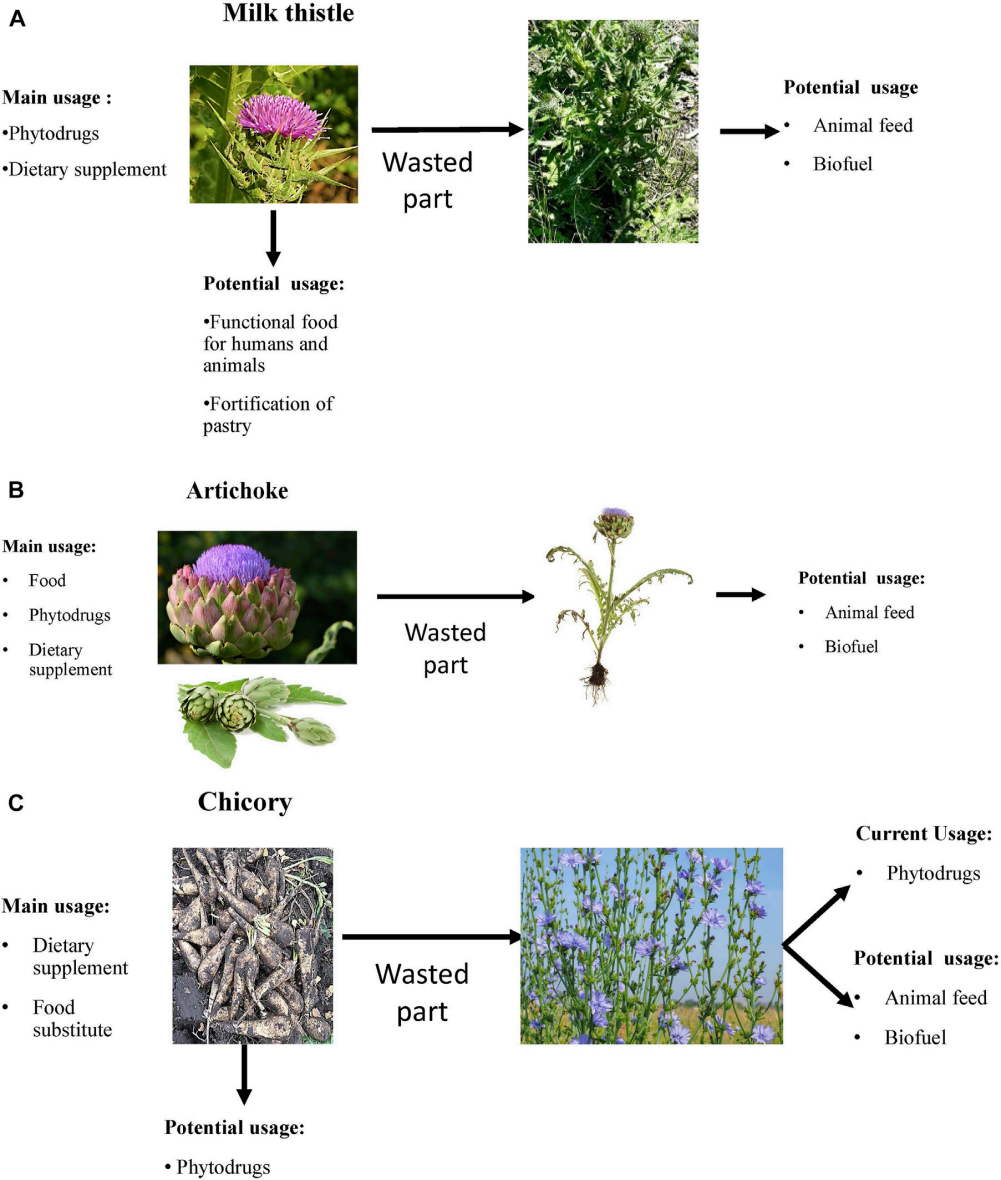
The rational use of plants with hepatoprotective properties. **(A)** Milk thistle; **(B)** Artichoke; **(C)** Chicory.

A significant part of the artichoke is wasted after the food industry uses artichoke flowers. The alternative use of the artichoke aerial part is presented in Figure 1. It has been proposed to use remaining lignocellulosic biomass as solid biofuel for heating houses or energy generation (Fernández et al., 2006; Barbosa et al., 2020). Several studies evaluated the heating value and combustion of artichoke biomass (Fernández et al., 2006; Oliveira et al., 2012; Gominho et al., 2018). Artichoke biomass and oil can be used for biodiesel production (Fernández et al., 2006). A bioplastic prepared from a plant’s leaves can potentially replace slowly degrading plastic (Mirpoor et al., 2022). Gominho et al. suggested using artichoke stalks as a source for paper production (Gominho et al., 2001). It appears that the artichoke has good potential to be used from stem-to-stern in food, pharmaceutical, and bioenergetic industries. The use of waste by food industry by-products could have a significant impact on the environment.

## Chicory

Chicory, *Cichorium intybus* L., a member of the Asteraceae family, is a well-known plant cultivated in Europe, Asia, Canada, the eastern part of the United States, northern Africa, and Australia (Kandeler and Ullrich, 2009). Practically, all plant parts are used as a source of food, phytodrugs, and dietary supplements (Perovic et al., 2021) (Table 1). In the past, fresh plant roots and young leaves were used as vegetables (Kandeler and Ullrich, 2009; Al-Snafi, 2016; Perovic et al., 2021). The plant was used to treat pulmonary and reproductive systems diseases, the biliary tract, hepatic disorders, diarrhea, and cancer (Al-Snafi, 2016). The hepatoprotective activities of chicory have been established in animals and humans (Sama et al., 1976; Saxena and Garg, 1979; Thabrew et al., 1982; Cimen et al., 2020). Inulin, a chicory root constituent, is extremely popular among consumers as a prebiotic and source of dietary fiber (Shoaib et al., 2016). It is sold as a dietary supplement worldwide. Additionally, chicory root and root bioactive constituent, inulin, are often used in the food industry as a coffee substitute or substitute for fat and sugar in pastry and ice cream (Kandeler and Ullrich, 2009; Shoaib et al., 2016; Wu and Cadwallader, 2019).

### Chicory Bioactive Constituents and Their Biological Activity

The chicory plant contains numerous biologically active constituents, including inulin, sesquiterpene lactones (e.g., chicoroisides B and C, sonchuside C), flavonoids, alkaloids, caffeic acid derivatives (e.g., chicoric acid, chlorogenic acid), vitamins E, β-carotene, and minerals (calcium, phosphorus, magnesium, and potassium) (Al-Snafi, 2016; Perovic et al., 2021) (Table 4). The distinct parts of the plant may contain different amounts of bioactive constituents. Plant roots are enriched in inulin and tannins but contain low amounts of phenolic acids (Al-Snafi, 2016). Inulin comprises ∼70% of constituents present in the fresh chicory root (Nwafor et al., 2017). The leaves and seeds contain high levels of phenolic compounds and flavonoids and low levels of inulin (Al-Snafi, 2016). Both wild and cultivated chicory is used in the food and dietary supplement industries. The aerial part of the plant may be used as a raw material for hepatoprotective phytodrugs, while the food and dietary supplement industries use roots.

**TABLE 4:** Phytochemical composition and pharmacological properties of chicory.

Plant name/part of the plant commonly used for medicinal purposes	Bioactive constituents	Pharmacological properties
**Chicory** (*Cichorium intybus* L.) Aerial part and roots 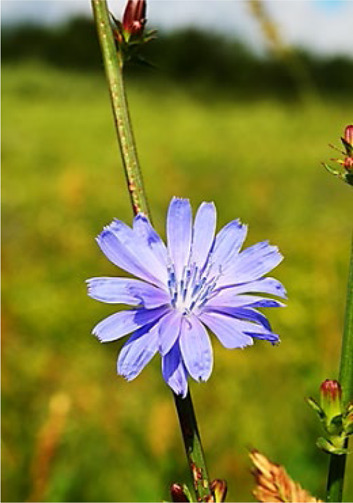 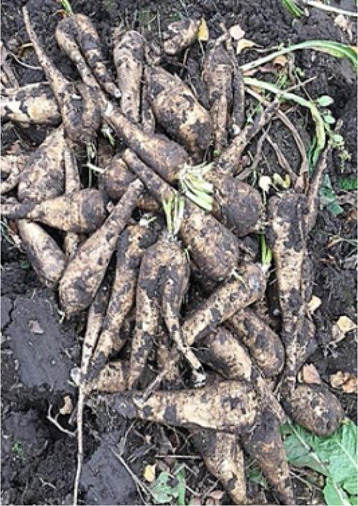	Chicoric acid 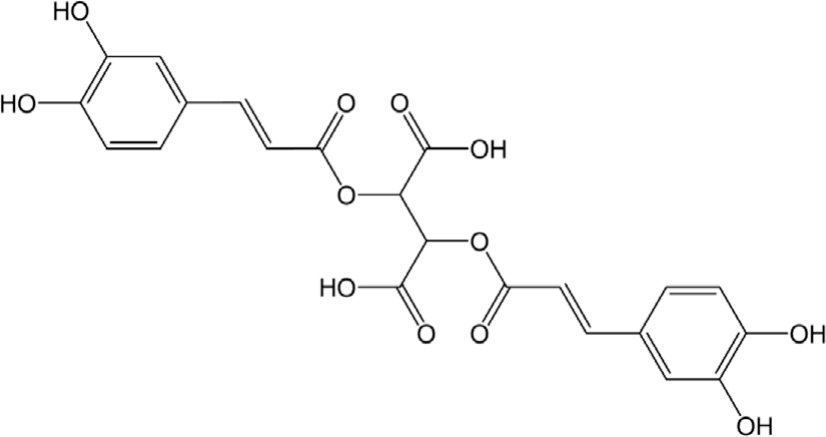 Esculetin 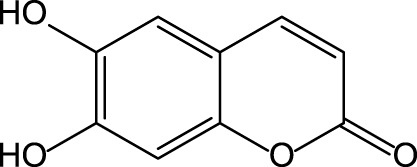 Cichoriin 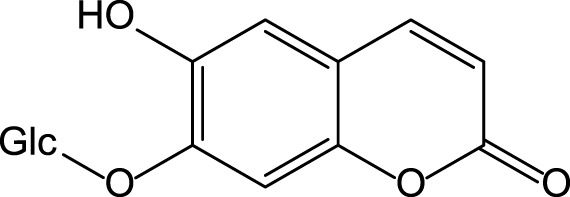 Caffeic acid 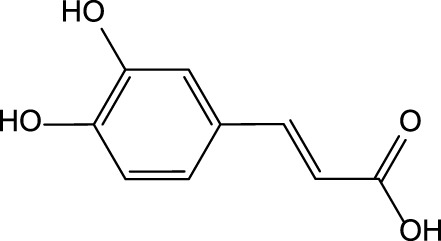 Chlorogenic acid 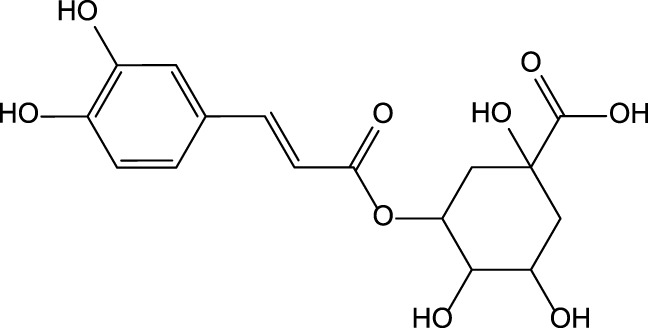 Caftaric acid 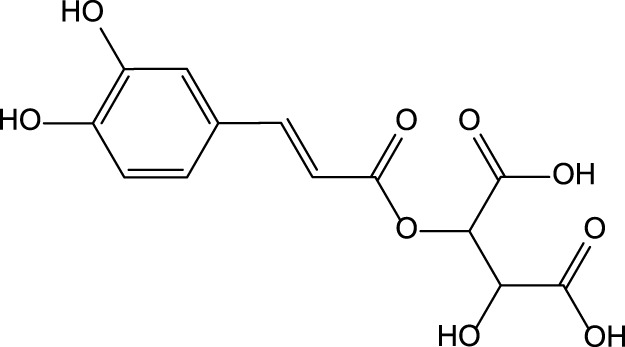	✓Hepatoprotective Ferenci et al. (1989); Huseini et al. (2005); Kolesnyk et al. (2009); Bortnikova et al. (2021) ✓Antibacterial/anthelmintic Perovic et al. (2021) Street et al. (2013) ✓Antiviral Janda et al. (2021) ✓Anti-inflammatory Huseini et al. (2005); Street et al. (2013) ✓Antioxidant Huseini et al. (2005);Street et al. (2013) ✓Gastroprotective Street et al. (2013) ✓Hypolipidemic Janda et al. (2021) ✓Immunomodulatory Huseini et al. (2005) ✓Anticancer Janda et al. (2021)

Chicory is reported to have several pharmacological properties, including antimicrobial, anthelmintic, antimalarial, anti-inflammatory, antioxidant, gastroprotective, and hepatoprotective (Street et al., 2013; Al-Snafi, 2016; Janda et al., 2021; Perovic et al., 2021) (Table 1). In addition, chicoric acid has hypoglycemic activity and antiviral effects (Zhang et al., 2014; Ferrare et al., 2018). Aqueous extracts from cultivated chicory possess antioxidant properties that were confirmed in animal models (Saybel', 2020). Hepatoprotective properties were reported for flavonoids isolated from *Cichorium glandulosum* Boiss. et Huet (Tong et al., 2015) (Table 4). Phenolic flavonoids extracted from leaves of chicory and artichoke also demonstrated hepatoprotective properties (Mohafrash and Mossa, 2020). The constituents present in the chicory extract are oxycoumarins: esculetin and cichoriin, hydroxycinnamic acids: chicory, chlorogenic, caftaric acids (Petropoulos et al., 2017; Saybel', 2020) (Table 4). These constituents have antioxidant and hepatoprotective properties. It is suggested that the complex biologically active constituents such as polyfructose, hydroxycinnamic acids, coumarins, and flavonoids, responsible for the plant’s hepatoprotective properties (Domitrovic and Potocnjak, 2016).

A double-blind, randomized controlled trial conducted in patients with chronic periodontitis has determined that treatment with 1 g of the chicory leaf extract decreases serum levels of total cholesterol, low-density lipoprotein cholesterol, and triglycerides, while increased serum levels of high-density lipoprotein cholesterol, uric acid, and total antioxidant capacity (Babaei et al., 2018). Additionally, the authors reported that the treatment decreased periodontal pocket depth.

Several clinical trials were conducted to evaluate inulin or chicory coffee’s effects on various human organ and system functions (e.g., lipid profile, bowel movement, and platelet aggregation Schumacher et al., 2011; Grela et al., 2014; Buddington et al., 2017). One of these studies evaluated whether chicory coffee has any beneficial effects on the cardiovascular system (Schumacher et al., 2011). The consumption of chicory coffee for 1 week resulted in a significant decrease in whole blood, red blood cell deformability, plasma viscosity, and macrophage migration inhibitory factors. A more recent randomized crossover trial has determined that supplementation of diets with chicory inulin contacting snack bars of people with low fiber intake led to an increase of beneficial members of the human intestinal microbiome, *Bifidobacterium* genus (Reimer et al., 2020).

### Chicory Hepatoprotective Properties

*Cichorium intybus* is a part of a phytodrug (Liv-52) introduced to the pharmaceutical market by the Himalaya Drug Company in 1955 (Himalaya Wellness Company., 2021b). Liv-52 includes *Capparis spinosa, Cichorium intybus, Solanum nigrum, Cassia occidentalis, Terminalia arjuna, Achillea millefolium,* and *Tamarix gallica* (Huseini et al., 2005; Himalaya Wellness Company., 2021b). Currently, Liv-52 and its modifications are widely used as dietary supplements in the United States and European countries. Liv-52 prevents the loss of functional integrity of the cell membrane, maintains cytochrome P-450 activity, and promotes hepatocellular regeneration (Himalaya Wellness Company., 2021b). The high hepatoprotective properties of this phytodrug were confirmed in patients with viral hepatitis, alcoholic and fatty liver, cirrhosis, anorexia (Sama et al., 1976; Huseini et al., 2005). Patients receiving Liv-52 for six consecutive months in a double-blind placebo-controlled trial had decreased ascites and significantly lower ALT and AST levels (Huseini et al., 2005). Huseini et al. attributed the hepatoprotective properties of the phytodrug to its antioxidant, anti-inflammatory, and immunomodulatory properties (Huseini et al., 2005). Recently, it has been proven that Liv-52 could treat ischemia reperfusion-induced liver damage in rats (Cimen et al., 2020).

Another clinical trial reported that supplementation of the diets of patients with non-alcoholic fatty liver disease for 12 weeks with a mixture of turmeric and chicory seeds resulted in a significant decrease of participants’ BMI and waist circumference and levels of serum alkaline phosphatase (Ghaffari et al., 2019).

We recently presented data demonstrating hepatoprotective properties of standardized dry extract obtained from the aerial part of wild chicory (*Cichorium intybus* L., DEC) (Bortnikova et al., 2021). DEC was developed at the VILAR. The analysis of the chemical composition revealed that the primary constituents present in DEC are phenol carboxylic acids [e.g., esters of caffeic, ferulic, coumaric acids with organic acids (quinic and tartaric)], flavonoids (isoquercetin, astragalin, rutin, luteolin, and kaempferol), and oxycoumarins (esculetin, cichoriin) (Saybel', 2020). The DEC contains 9.20 ± 0.43% of phenolic constituents calculated as chicoric acid.

DEC has significant hepatoprotective properties confirmed in a model of acute toxic hepatitis induced by a single subcutaneous injection of mercuric chloride (HgCl_2_). Mercuric chloride is a potent thiol poison leading to protein structure damage and inhibition of thiol-containing antioxidants and liver damage caused by the development of oxidative stress (Deng et al., 2012).

Mercuric chloride intoxication led to increased relative liver weight in the HgCl_2_ treated group (Bortnikova et al., 2021). Rats treated with HgCl_2_ had a statistically significant increase in total protein, glucose, total cholesterol, total bilirubin, triglycerides, and the GGT, ALP, AST, and AST activities. The histological liver analysis showed the presence of hepatocyte dystrophy, characterized by hyaline droplet dystrophy. Treatment of rats with DEC in doses of 100 and 500 mg/kg for 3 weeks prevented a sharp decrease in the animals’ body weight and physical activity. The relative liver weight was lower than in animals treated with HgCl_2_ (Bortnikova et al., 2021). The administration of the DEC resulted in a decrease in the activity of several liver enzymes characterizing the functional state of the liver and contributed to experimental rats’ fast recovery (Bortnikova et al., 2021). These changes were more noticeable in animals receiving the maximal DEC dose. Lipid, protein, glucose, and bilirubin levels decreased significantly in the DEC groups compared to the HgCl_2_ group. The dystrophic changes in hepatocytes of rats treated with DEC 500 mg/kg were not visible. The comparison of hepatoprotective activity of DEC and Silimar indicates that the hepatoprotective effect of DEC at the dose 500 mg/kg was comparable with the effects observed in the group receiving 100 mg/kg of Silimar (Bortnikova et al., 2021). Preclinical data demonstrating DEC hepatoprotective properties in rats need to be confirmed in clinical studies.

Additionally, we have reported that DEC has immunomodulatory properties (Saybel', 2020). Oral administration of DEC to immunosuppressed mice at a 50 mg/kg dose for five consecutive days resulted in upregulation of innate, humoral, and cell-mediated immune responses. Similar upregulations were not observed in animals with normal immune responsiveness.

### Chicory-Based Functional Food and Supplements

The food and dietary supplement industries actively use chicory roots (e.g., inulin and oligofructose) as a food substitute, dietary, functional, or food supplements (Wu and Cadwallader, 2019) (Table 1). Inulin is widely used as a prebiotic supporting the intestinal microbiome, specifically stimulating the development of the bifidobacteria in the colon (Niness, 1999). Additionally, inulin acts as dietary fiber (Niness, 1999). Therefore, inulin consumption may increase intestinal peristalsis and may help people experiencing constipation.

Overconsumption of fatty and sugary food has contributed to the disproportional increase in the number of people who are obese or have diabetes. Therefore, reducing fat and sugar in food products could help reduce this trend. Moreover, many people are interested in a healthy lifestyle and healthy eating habits. When dissolved in the water or milk, inulin forms a creamy texture and gives the food a fatty feel (Niness, 1999). Oligofructose has a sweet taste and can be used as a sugar substitute (Niness, 1999). Replacement of high-fat milk and sugar with inulin and oligofructose in milk products (e.g., ice cream) may significantly reduce fat and sugar consumption. At the same time, it will not compromise product taste. Significantly, inulin and oligofructose do not influence glucose levels and insulin secretion (Niness, 1999).

### Animal Feed

Lastly, the aerial part and root of chicory can be used as fodder and complementary treatment of livestock (Nwafor et al., 2017) (Table 1). It appears that chicory extracts or phytochemicals like inulin and volatile oils can eliminate or suppress the growth of intestinal worms and other parasites found in animals (Githiori et al., 2006). Therefore, fodder fortified by chicory foliage can promote overall livestock health and prevent intestinal parasite development. Adding 0.1% of chicory powder to the broiler’s diet increased body weight but significantly lower abdominal fat compared to control birds (Khoobani et al., 2020). The same study reported that adding probiotics or chicory to the diet improved the broiler’s ileal microbiome. When chicory is given in excessive amounts to farm animals, it can negatively affect the growth and performance of the livestock.

## Discussion

This review article has focused on the complex use of distinct parts of MT, artichoke, and chicory for medicinal purposes, food for humans and animals, and potentially biofuel. As presented in the preceding sections, all parts of these plants can potentially be used as phytodrugs, dietary supplements, components of functional food, and food for humans and animals. It is important to note that usually, only one part of these plants is actively used by the food or drug/dietary supplement industry, and as a result, dozens of articles focus on the biological properties of this part of the plant and constituents responsible for its activity. There are very few articles analyzing real-world aspects of by-product use. Review articles summating available literature on by-product use cite 1–2 articles or state that based on the known biological activity of by-product, it can be used as a dietary supplement, animal feed, or biofuel.

The actively used part of MT is plant fruit (e.g., whole fruit, seeds, oilseed cake, or oil) (Figure 1). It is actively used for medicinal purposes to produce phytodrugs and dietary supplements but can also be added to bakery products. The fortification of the bread with MT oil and oilseed cake improves the bread quality and may help support proper liver function and provide an additional source of amino acids, minerals, and vitamins. Several reports demonstrate the benefits of oilseed cake as a supplement in improving the wellbeing and survival of farm animals (see Section 3.4). The question then becomes what happens to the remaining parts of the plant. Are they wasted? MT leaves are occasionally used to prepare salads. The addition of the aerial part of MT to animal feed may have nutritional benefits, helping to improve the weight gain and survival of farm animals (Andrzejewska et al., 2015; Bagno et al., 2020). It is doubtful that the aerial part of the plant will be used as the main feed for animals due to its low nutritional value (Stastnik et al., 2020). The aerial part represents 70–80% of the whole plant and can be potentially used as a biofuel source.

The food industry uses flowers of artichoke (Figure 1). It appears that 80% of the plant is wasted. Artichoke leaves demonstrate many biological activities, including hepatoprotective. Therefore, they can be used for medicinal purposes as a phytodrug or dietary supplement. It has been proposed that the aerial part of artichoke can be used as animal feed and biofuel.

The most used part of chicory is the root (Figure 1). Roots from cultivated chicory are inulin’s core sources, actively used as dietary supplements and substitutes for fat and sugar. The aerial part of the plant is usually wasted. Bioactive constituents present in the aerial part of the plant possess hepatoprotective properties. The aerial part of the cultivated plant can serve as a raw material for the pharmaceutical or dietary supplement industry, and potentially, as biofuel.

As another example of a plant with the predominant use of roots is licorice (*Glycyrrhiza glabra*)*.* It is a cough remedy (Kamei et al., 2003; Nosalova et al., 2013; Ruetzler et al., 2013; Kuang et al., 2018) and a gum flavor for candies and food (Kwon et al., 2020). It can treat digestive problems, atopic dermatitis, bacterial and viral infections (Kwon et al., 2020). The licorice roots also possess hepatoprotective properties (Li et al., 2019). It has been reported that licorice ethanolic extract prepared in the leaves possesses antimicrobial activity against Gram-positive bacteria, which was higher than in extract prepared from roots (Irani et al., 2010). Compounds isolated from the leaves of *G. uralensis* demonstrated anti-inflammatory properties *in vitro* (Wang et al., 2019).

One of the best examples is the stem-to-stern use of pumpkin (*Cucurbita pepo*) and corn (*Zea mays*). Pumpkin is a well-known source of food for humans and farm animals. It is highly nutritional (Klyuchnikova et al., 2011; Brennan, 2020). Pumpkin is rich in proteins, carbohydrates, dietary fiber, vitamin A, vitamin C, magnesium, and potassium (Brennan, 2020). All parts of the pumpkin are edible, including seeds, seed oil, and even leaves. Pumpkins can be given to domestic animals to support their digestive system (Lans, 2019). Elevated levels of α-carotene, β-carotene and β-cryptoxanthin, well-known antioxidants, and vitamin C could help to improve immune system functions and protect against infections, reduce risk of cancer development (Ben-Amotz and Fishier, 1998; Zhou et al., 2016).

Corn is high in dietary fiber, regulates bowel movement, prevents constipation, and decreases cholesterol and glucose levels (Cooper et al., 2017). Various parts of corn are used as a human food source (ear of corn, corn flour, cornmeal, and high-fructose corn syrup), food for farm animal food (plant), a source for fuel, ethanol, and plastic (Fowler, 2012; Gwirtz and Garcia-Casal, 2014). Corn constituents are also used as dietary supplements. For example, corn contains inositol hexaphosphate (IP6), a phosphorylated carbohydrate that has anti-tumor properties and modulatory effects on macrophages (Wee et al., 2021).

The potential of the whole plant use for MT, artichoke, chicory, and other plants is evident. Unfortunately, different industries use distinct parts of the plant (Figure 1). Other parts of the plant are likely wasted. Considering the growing human population, responsible management of plant resources could provide sufficient medicine and food supplies for humans and animals.

The whole plant use should be a priority for businesses and the government. For example, when chicory plant is collected at the field, roots can be sold to the food industry and aerial part to pharmaceutical/dietary supplement companies. Alternatively, dietary supplement companies producing inulin from roots can use the aerial part of the plant to produce supplements supporting liver function.

We feel that the research community needs to analyze and summarize the potential use of distinct parts of plants. This includes estimating the economic judiciousness and proposing a complex and rational use plan for plants to pharmaceutical/dietary supplements, food industries, and country government. The joint efforts with the government must stimulate companies to utilize by-products coming from their technological processes by providing, for example, tax credits.

Many plants used by pharmaceutical and dietary supplement companies are wild plants. About 20 North American medicinal plants are at risk of endangerment due to overharvesting and decreased natural habitats (United Plant Savers, 2021). The development of new plant cultivation techniques could help overcome the overharvesting of precious plants.

## Conclusion

Milk thistle, artichoke, and chicory are highly versatile and valuable plants that can be potentially used as phytodrugs, dietary supplements, functional food, food for humans and animals, or biofuel. Distinct parts of these plants are used by pharmaceutical/dietary supplement and food industries. Other parts of plants are possibly wasted. These are only three examples of the potential use of the whole plant. There are many other plants, some parts of which are used, and others are wasted. A plan for the rational stem-to-stern use of the whole plant needs to be developed for most, if not all, plants actively used by food or drug/supplement industries. This should include measures to prevent wild plant overharvesting and the active introduction of cultivation techniques utilizing all plant parts.

## Abbreviations

ALT, alanine aminotransferase; ALP, alkaline phosphatase; AST, aspartate aminotransferase; CCl4, carbon tetrachloride; DEC, dry extract obtained from wild chicory; DSHEA, dietary supplement health and education act; GGT, γ-glutamyl transferase; ERK1/2, extracellular signal-regulated kinase 1/2; HCV, hepatitis C virus; HgCl2, mercury chloride; IL1α, interleukin 1 alpha; IP6, inositol hexaphosphate; MT, Milk Thistle; NF-kB, nuclear factor kappa B; RNA, ribonucleic acid; STAT-1, signal transducer and activator of transcription 1; STAT-3, signal transducer and activator of transcription 3; TNFα, tumor necrosis factor-alpha; US FDA, the United States food and drug administration; VILAR, All-Russian research institute of medicinal and aromatic plants.
